# REDD1 deletion and treadmill running increase liver hepcidin and gluconeogenic enzymes in male mice

**DOI:** 10.1017/jns.2023.37

**Published:** 2023-04-14

**Authors:** David E. Barney, Bradley S. Gordon, Stephen R. Hennigar

**Affiliations:** 1 Department of Nutrition & Integrative Physiology, Florida State University, Tallahassee, FL, USA; 2 Pennington Biomedical Research Center, Baton Rouge, LA, USA

**Keywords:** Exercise, Gluconeogenesis, Hepcidin, Iron deficiency, REDD1, CREB3L3, hepatic-specific cAMP response element binding protein-3-like-3, CRP, C-reactive protein, IL, interleukin, KO, knockout, PGC1α, peroxisome proliferator-activated receptor-γ coactivator-1α, REDD1, regulated in development and DNA damage response-1, qRT-PCR, quantitative real-time polymerase chain reaction, WT, wild-type

## Abstract

The iron-regulatory hormone hepcidin is transcriptionally up-regulated by gluconeogenic signals. Recent evidence suggeststhat increases in circulating hepcidin may decrease dietary iron absorption following prolonged exercise, however evidence is limited on whether gluconeogenic signals contribute to post-exercise increases in hepcidin. Mice with genetic knockout of regulated in development and DNA response-1 (REDD1) display greater glycogen depletion following exercise, possibly indicating greater gluconeogenesis. The objective of the present study was to determine liver hepcidin, markers of gluconeogenesis and iron metabolism in REDD1 knockout and wild-type mice following prolonged exercise. Twelve-week-old male REDD1 knockout and wild-type mice were randomised to rest or 60 min treadmill running with 1, 3 or 6 h recovery (*n =* 5–8/genotype/group). Liver gene expression of hepcidin (*Hamp*) and gluconeogenic enzymes (*Ppargc1a*, *Creb3l3*, *Pck1*, *Pygl*) were determined by qRT-PCR. Effects of genotype, exercise and their interaction were assessed by two-way ANOVAs with Tukey's *post-hoc* tests, and Pearson correlations were used to assess the relationships between *Hamp* and study outcomes. Liver *Hamp* increased 1- and 4-fold at 3 and 6 h post-exercise, compared to rest (*P*-adjusted < 0⋅009 for all), and was 50% greater in REDD1 knockout compared to wild-type mice (*P* = 0⋅0015). Liver *Ppargc1a*, *Creb3l3* and *Pck1* increased with treadmill running (*P* < 0⋅0001 for all), and liver *Ppargc1a*, *Pck1* and *Pygl* were greater with REDD1 deletion (*P* < 0⋅02 for all). Liver *Hamp* was positively correlated with liver *Creb3l3* (*R* = 0⋅62, *P* < 0⋅0001) and *Pck1* (*R* = 0⋅44, *P* = 0⋅0014). In conclusion, REDD1 deletion and prolonged treadmill running increased liver *Hamp* and gluconeogenic regulators of *Hamp*, suggesting gluconeogenic signalling of hepcidin with prolonged exercise.

## Introduction

Iron metabolism is systemically regulated by the hormone hepcidin, encoded by the hepcidin antimicrobial peptide (*HAMP*) gene^(1,2)^. Hepcidin is secreted by the liver to negatively regulate cellular iron efflux by binding to, occluding and signalling for the lysosomal degradation of ferroportin, the only known mammalian cellular exporter of iron^(3,4)^. Through hepcidin-mediated degradation of ferroportin, iron is sequestered out of circulation and into tissues, and dietary iron is prevented from export out of absorptive enterocytes, thus inhibiting dietary iron absorption. The two canonical pathways of hepcidin production are through transcriptional regulation of *HAMP* by (1) iron-related signals through the BMP–SMAD pathway and (2) inflammation through interleukin-6 (IL-6) and the JAK–STAT pathway. More recent evidence indicates that hepcidin is produced in response to gluconeogenic signals, whereby *HAMP* transcription is promoted by peroxisome proliferator-activated receptor-γ coactivator-1α (PGC1α) and hepatic-specific cAMP response element binding protein-3-like-3 (CREB3L3)^(5)^. A limited number of studies support these findings and demonstrate increases in hepcidin and impacts on iron regulation in response to conditions resulting in increased gluconeogenesis, such as decreased food intake or increased physical activity^(6–12)^.

The literature consistently reports a peak in circulating hepcidin 3–6 h following a bout of prolonged exercise, which is suggested as a reason for the decline in iron status often seen in physically active populations^(13,14)^. A recent study in trained female and male collegiate cross country runners reported increased hepcidin and decreased dietary iron absorption following a bout of prolonged running^(15)^. In the present study, the reason for the increase in hepcidin with exercise was not clear, but may have been due to increases in IL-6^(16)^. An alternative explanation is PGC1α–CREB3L3 promotion of hepcidin in response to gluconeogenesis during the prolonged run. Pasiakos *et al.*^(8)^ reported that circulating hepcidin following a 4-d military training operation was positively correlated with severity of energy deficit. Another study from our group showed that performing strenuous exercise in a 45% energy deficit exacerbated the post-exercise hepcidin response and reduction in dietary iron absorption compared to exercise in energy balance^(11)^. Despite these clinical findings, evidence is limited on gluconeogenic signalling of *HAMP* in the liver following a bout of prolonged exercise.

Regulated in development and DNA damage response-1 (REDD1, also known as DDIT4) is a ubiquitously expressed stress-response protein, encoded by *REDD1*. REDD1 quickly responds to various cellular and energetic stresses, including acute fasting and prolonged exercise^(17)^. REDD1 is implicated in a number of metabolic roles, namely as an inhibitor of Akt–mammalian target of rapamycin complex-1 signalling and as an enhancer of autophagy, through which REDD1 is thought to decrease protein anabolism and increase substrate availability during periods of energetic stress^(18,19)^. REDD1 also has glucoregulatory roles, possibly as a mediator of glucocorticoid signalling^(20–23)^. Accordingly, REDD1 deletion results in greater energetic distress in mice in response to prolonged exercise. In skeletal muscle of mice following 90 min treadmill running, Britto *et al.*^(18)^ reported greater phosphorylation of adenosine monophosphate protein kinase A and greater glycogen depletion in skeletal muscle of REDD1 knockout (KO) mice compared to wild-type (WT) controls. Given these findings, REDD1 deletion may result in greater hepatic gluconeogenesis following prolonged treadmill running and provide a novel model to explore gluconeogenic regulation of hepcidin. Therefore, the primary aim of the present study was to determine the impact of REDD1 deletion on the hepcidin response to a prolonged bout of treadmill running and assess corresponding gluconeogenic signals (e.g., PGC1α, CREB3L3). We hypothesised that liver *Hamp* would increase following exercise with a greater increase in REDD1 KO mice compared to WT mice, which would coincide with increased expression of gluconeogenic regulators of *Hamp* and genes for enzymes indicating hepatic gluconeogenesis.

## Methods

This was a secondary analysis of a study that sought to determine the impact of REDD1 deletion on skeletal muscle gene expression following exercise^(21)^. Animal research was approved by the Animal Care and Use Committee at the Pennsylvania State University College of Medicine, and this study adhered to the ARRIVE Guidelines for Reporting Animal Research.

### Animals and diet

Twelve-week-old male REDD1 KO and WT mice were used for all experiments^(21)^. Permission to use REDD1 KO mice was granted by Dr. Elena Feinstein (Quark Pharmaceuticals; Fremont, CA, USA). Breeding pairs on a B6/129F1 background were acquired from Dr. David Williamson (Pennsylvania State University – Harrisburg) and were bred in the Pennsylvania State University College of Medicine animal facility. Age-matched WT B6/129F1 mice were acquired from Taconic Biosciences (Hudson, NY, USA). Mice were single-housed in a temperature- and light-controlled vivarium (25°C, 12:12 h light:dark cycle) with *ad libitum* access to water and food. Mice were fed chow diets with ~200 mg/kg iron (Table 1).

**Table 1.** Nutritional composition of diets fed to REDD1 KO and WT mice^a^

Component	NIH #31M	2018 Teklad
Energy and macronutrients		
Energy density (kcal/g)	3⋅2	3⋅1
Carbohydrate (%)	65	44
Fat (%)	14	6⋅2
Protein (%)	21	18
Minerals		
Iron (mg/kg)	206	200
Calcium (mg/kg)	1100	10 000
Copper (mg/kg)	17	15
Iodine (mg/kg)	1⋅97	6⋅0
Magnesium (mg/kg)	220	2000
Manganese (mg/kg)	150	100
Phosphorus (mg/kg)	930	7000
Potassium (mg/kg)	570	6000
Sodium (mg/kg)	280	2000
Zinc (mg/kg)	58	70
Vitamins		
Vitamin A (IU/g)	26	15
Thiamine (mg/kg)	77	17
Riboflavine (mg/kg)	7⋅6	15
Niacin (mg/kg)	83	70
Pantothenic acid (mg/kg)	38	33
Pyridoxine (mg/kg)	11	18
Biotin (mg/kg)	0⋅29	0⋅40
Folate (mg/kg)	1⋅6	4⋅0
Vitamin B_12_ (μg/kg)	56	80
Vitamin D (IU/g)^b^	4⋅1	1⋅6
Vitamin E (mg/kg)	47	74
Vitamin K (mg/kg)^c^	22	50
Choline (mg/g)	1⋅9	1⋅2

a The NIH #31M diet was fed to WT mice by Taconic Biosciences. The 2018 Teklad global 18% protein chow diet was fed to WT mice upon delivery to the laboratory at ~10 weeks of age and to REDD1 KO mice throughout their lifespan.

b Vitamin D is given as Vitamin D_3._

c Vitamin K is given as menadione (Vitamin K_3_).

### Treadmill acclimation, running protocol and tissue collection

**Fig. 1.**
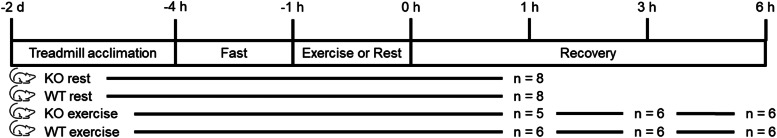
Protocol overview and sample sizes of REDD1 KO and WT mice with outcomes measured after rest or 1, 3 or 6 h recovery from 60 min treadmill running. At 12 weeks of age, male REDD1 KO and WT mice completed a treadmill acclimation once per day for the 2 d prior to the experimental protocol. Food was removed from cages 3 h prior to experimental protocols. The exercise protocol consisted of a 10 min warm-up (5 min at 10 m/min and 5 min at 15 m/min) immediately followed by 50 min at 18 m/min (~60% VO_2_max), all performed at 5% incline. Exercised mice were euthanized at 1, 3 and 6 h post-exercise. Rested mice were placed in cages next to moving treadmills for the protocol duration and euthanized at the 1 h timepoint. KO, knockout; REDD1, regulated in development and DNA damage response-1; WT, wild-type.

Mice were acclimated to a Columbus Instruments rodent treadmill (Columbus, OH, USA) over the 2 d immediately preceding experiments (Fig. 1)^(21)^. Acclimation was performed at a 5% incline and consisted of 10 min at 10 m/min, 5 min at 15 m/min and 5 min at 18 m/min performed once per day between 08.00 and 09.00 at the start of the light cycle. Following the 2 d acclimation, mice were randomised indiscriminate of physical characteristics (body size, exercise capacity, etc.) to rest (no exercise) or treadmill running (exercise) and tissues were collected 1, 3 or 6 h post-exercise. Sample sizes for REDD1 KO mice were *n =* 8 (rest), 5 (1 h), 6 (3 h) and 6 (6 h); sample sizes for WT mice were *n =* 8 (rest), 6 (1 h), 6 (3 h) and 6 (6 h).

On experimental days, food was removed 3 h prior to experimentation while water was accessible *ad libitum*. Food deprivation started at the onset of the light cycle. The 3 h fast was initially designed to partially normalise food intake while avoiding the dramatic impact of a prolonged overnight fast on REDD1-related signalling pathways^(24)^, though this likely served a similar purpose for genes that increase with fasting and impact iron homeostasis^(5)^. The treadmill run began with a 10 min warm-up (5 min at 10 m/min and 5 min at 15 m/min) immediately followed by 50 min at 18 m/min, all at a 5% incline. This maximum speed corresponds to an intensity of ~60% VO_2_max in mice^(25)^. This protocol has been previously shown to produce transient increases in REDD1 gene and protein expression in the plantaris muscle of these mice 1 h following exercise, with gene and protein expression equivalent to rest at 3 and 6 h post-exercise^(21)^. Similar protocols have also been used to increase *Redd1* and *Hamp* expression in mouse livers^(26,27)^. After exercise completion, mice were returned to their cages, given *ad libitum* access to water only, and euthanized 1, 3 or 6 h later. Mice in the rested groups were placed in cages next to the treadmill for 1 h and were euthanized 1 h later. Mice were anesthetised with isoflurane (2–3%) and tissues were excised, flash frozen in liquid nitrogen and stored at –80°C until analysis. Liver and spleen tissue were used for the present study.

### Quantitative real-time polymerase chain reaction (qRT-PCR)

Using qRT-PCR, gene expression of *Redd1*, *Hamp*, PGC1α (*Ppargc1a*), *Creb3l3*, phosphoenolpyruvate carboxykinase (*Pck1*), glycogen phosphorylase (*Pygl*), IL-6 (*Il6*), C-reactive protein (*Crp*), α-1-acid glycoprotein (*Orm1*) and serum amyloid A1 (*Saa1*) were assessed in liver, and erythroferrone (*Erfe*) was assessed in spleen. RNA was isolated from liver using TRIzol (Invitrogen; Waltham, MA, USA). Splenic RNA was isolated using the Direct-zol RNA Microprep kit (Zymo Research; Irvine, CA, USA). RNA was reverse-transcribed into cDNA with the High-Capacity cDNA Reverse Transcription kit (Applied Biosystems; Foster City, CA, USA). RNA and cDNA quantity and quality (A260/280) were assessed by a NanoDrop One spectrophotometer (ThermoFisher Scientific; Waltham, MA, USA). Liver RNA and cDNA absorbance ratios (mean ± sd) were 2⋅04 ± 0⋅03 and 1⋅85 ± 0⋅01, and spleen RNA and cDNA absorbance ratios were 1⋅96 ± 0⋅05 and 1⋅81 ± 0⋅02, respectively. qRT-PCR was performed in a QuantStudio-3 PCR instrument (ThermoFisher Scientific) using TaqMan Fast Advanced Master Mix (ThermoFisher Scientific) with TaqMan primer-probe sets for all genes (Supplementary Table S1) except *Redd1*, which was assessed with primers previously reported by Gordon *et al.*^(21)^ and PowerUp SYBR Green Master Mix (ThermoFisher Scientific, Supplementary Table S2). Gene expression data were normalised to the housekeeper gene β-actin (*Actb*), which was unaffected by genotype or exercise (*P* > 0⋅88 for all). The following equation was used to determine a change in cycle quantification (*Cq*): Δ*Cq* = *Cq*_Gene_ – *Cq*_Housekeeper_. Using mean Δ*Cq* of rested WT mice for baseline, fold change was calculated using the ΔΔ*Cq* method.

### Liver and spleen non-heme iron

Liver and spleen non-heme iron concentrations were determined as previously described^(28)^. Briefly, samples were homogenised in protein precipitation solution (0⋅53 N HCl, 5⋅3% trichloroacetic acid) using a bead-mill, boiled at 95°C for 30 min, and centrifuged at 21 000×*g* for 10 min. Non-heme iron concentrations were analysed in supernatants by colorimetric spectrophotometry using the Iron-SL kit (Sekisui Diagnostics; Burlington, MA, USA).

### Statistical analysis

Prior to tissue analysis for this secondary analysis, data from Banzet *et al.*^(27)^ were used for a power analysis in G*Power version 3.1^(29)^. A total sample size of twenty animals was estimated to give >95% power to detect an increase in liver *Hamp* with treadmill running in three post-exercise groups, compared to a rest group (Supplementary Table S3), which was adequate for both WT (*n =* 26) and KO mice (*n =* 25). Statistical analysis was performed in GraphPad Prism version 9.3 (San Diego, CA, USA). Normality was assessed using the Shapiro–Wilk test. If normality was rejected (*P* ≥ 0⋅05), data were log-transformed to create a normal distribution. The effect of exercise on liver *Redd1* expression in WT mice was assessed by a one-way analysis of variance with Tukey's honest significant difference test for multiple comparisons, and the effects of REDD1 genotype and exercise on all other outcomes were assessed by two-way analyses of variance with main effects of genotype, exercise and the genotype-by-exercise interaction. If an interaction effect was significant, *post-hoc* multiple comparisons were made with Tukey's test. If the exercise effect was significant without a significant interaction effect, one-way analyses of variance were performed with Tukey's test to assess differences between exercise groups collapsed across genotypes. Mean and least-squared mean differences are reported with 95% CIs in parentheses. To explore the relationships between liver *Hamp* and other outcomes, Pearson correlations were performed on data collapsed across genotypes and timepoints and are reported as Pearson's *R*. When log transformation did not create a normal distribution, Spearman correlations were performed on untransformed data and are reported as Spearman's rho (*ρ*). *A priori*, *α* was set to 0⋅05.

## Results

**Fig. 2.**
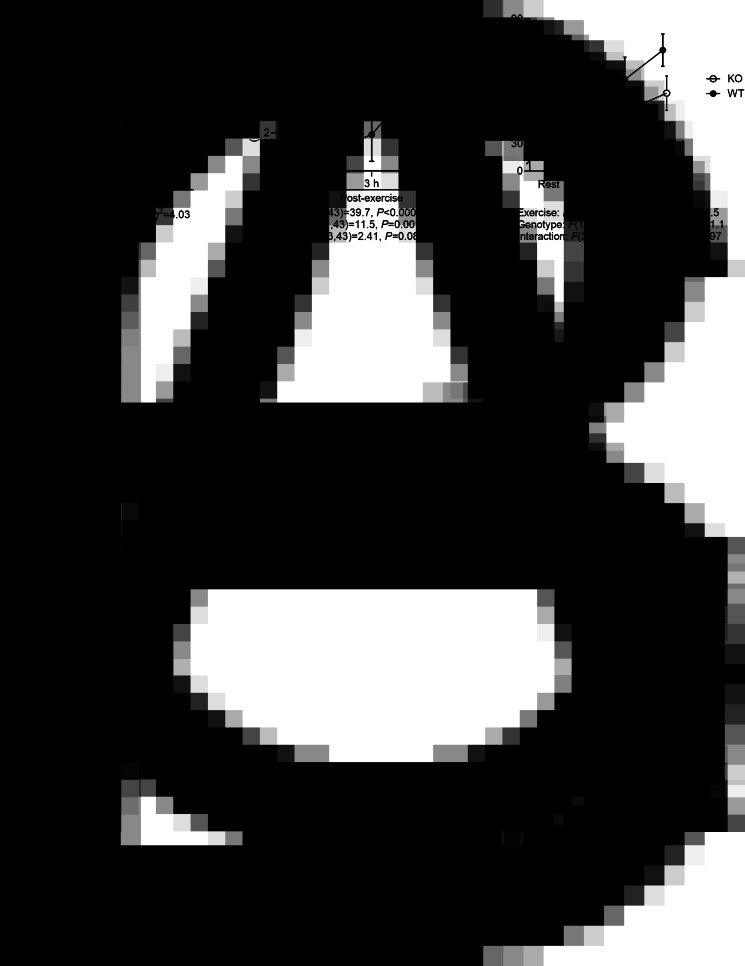
Liver expression of *Redd1* in WT mice (a) and liver expression of *Hamp* (b) and liver non-heme iron (c) in REDD1 KO and WT mice following rest or 1, 3 or 6 h recovery from 60 min treadmill running. Gene expression is fold change relative to rested WT mice. Liver *Redd1* expression was assessed by one-way analysis of variance with Tukey's *post-hoc* analysis. Liver *Hamp* expression and liver non-heme iron were assessed by two-way analyses of variance. Samples sizes are *n =* 8 per group for rested mice and *n =* 5–6 per group for exercised mice. Main effects of exercise, genotype and their interaction are presented as: *F*-value (degrees of freedom), *P*-value and *η*^2^. Different letters indicate a significant *post-hoc* difference within WT mice. Data are means (95% CIs). KO, knockout; REDD1, regulated in development and DNA damage-1; WT, wild-type.

In WT mice, liver *Redd1* increased 3-fold at 1 h post-exercise compared to rest [3⋅67 (0⋅598, 6⋅74), *P*-adjusted = 0⋅015] and returned to resting levels at 3 h post-exercise [−3⋅70 (−6⋅98, −0⋅413), *P*-adjusted = 0⋅024 compared to 1 h; −0⋅0275 (−3⋅10, 3⋅04), *P*-adjusted > 0⋅99] compared to rest (Fig. 2(a)). Liver *Redd1* expression at 6 h post-exercise was not different from any other timepoint [2⋅18 (−0⋅895, 5⋅25), *P*-adjusted = 0⋅023 compared to rest; –1⋅50 (−4⋅77, 1⋅79), *P*-adjusted = 0⋅60 compared to 1 h; 2⋅20 (−1⋅08, 5⋅48), *P*-adjusted = 0⋅27 compared to 3 h]. There was an effect of genotype for liver *Hamp* where expression was 50% greater in REDD1 KO mice compared to WT mice [0⋅976 (0⋅396, 1⋅56), *P* = 0⋅0015, Fig. 2(b)]. An effect of exercise was also observed for liver *Hamp* (*P* < 0⋅0001), where expression was not different at 1 h post-exercise compared to rest [−0⋅257 (−1⋅47, 0⋅955), *P*-adjusted = 0⋅94], but doubled at 3 h [1⋅48 (0⋅297, 2⋅66), *P*-adjusted = 0⋅0088] and increased nearly 4-fold at 6 h post-exercise [3⋅71 (2⋅53, 4⋅89), *P*-adjusted < 0⋅0001] compared to rest. Liver non-heme iron concentrations were 32% greater in WT mice compared to REDD1 KO mice [15⋅0 (9⋅27, 20⋅7) μg/g wet weight, *P* < 0⋅0001, Fig. 2(c)]. An effect of exercise was observed for liver non-heme iron (*P* = 0⋅0026), where concentrations increased 30% at 6 h post-exercise compared to rest [14⋅9 (2⋅19, 27⋅6) μg/g wet weight, *P*-adjusted = 0⋅016], but concentrations did not differ between any other timepoint [*P*-adjusted > 0⋅11 for all]. Splenic non-heme iron did not differ with genotype or exercise (*P* > 0⋅54 for all, data not shown), and splenic *Erfe* expression was not detected.

**Fig. 3.**
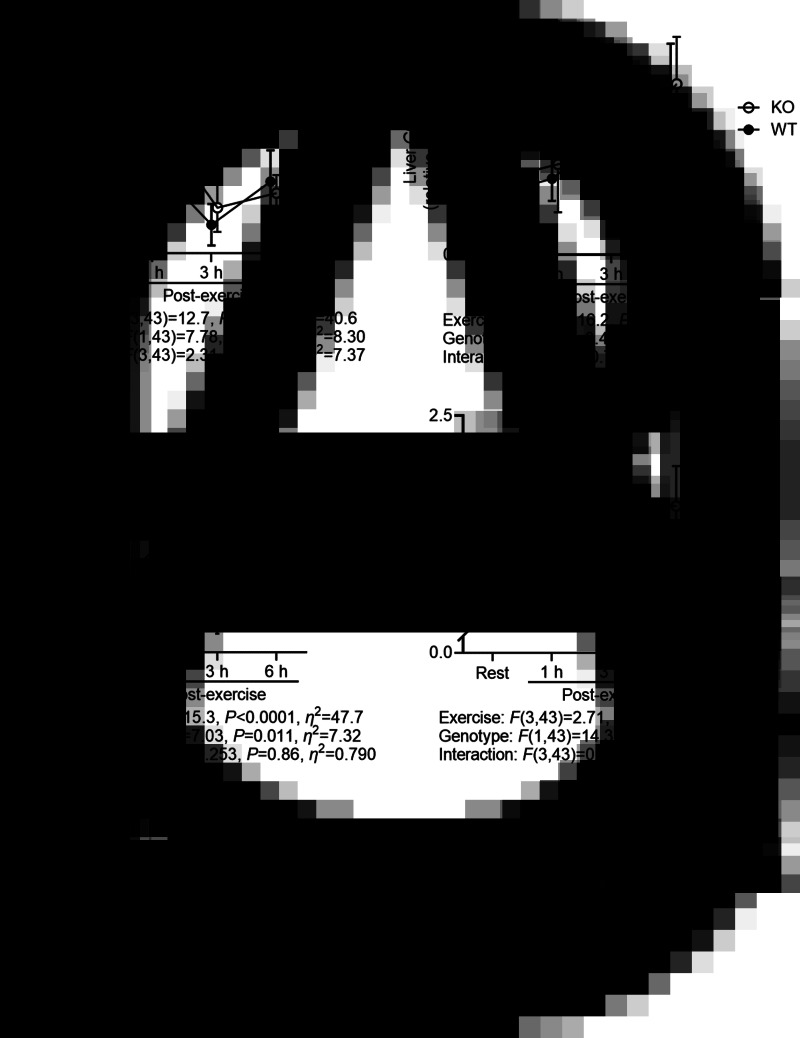
Liver gene expression of gluconeogenic regulators of *Hamp* (a, b) and gluconeogenic (c) and glycogenolytic enzymes (d) in REDD1 KO and WT mice following rest or 1, 3 or 6 h recovery from 60 min treadmill running. Gene expression is fold change relative to rested WT mice. Data were analysed using two-way analyses of variance. *Ppargc1a*, *Pck1* and *Pygl* were log-transformed for analysis. Samples sizes are *n =* 8 per group for rested mice and *n =* 5–6 per group for exercised mice. Main effects of exercise, genotype and their interaction are presented as: *F*-value (degrees of freedom), *P*-value and *η*^2^. Data are presented as untransformed means (95% CIs). KO, knockout; REDD1, regulated in development and DNA damage-1; WT, wild-type.

Markers of gluconeogenesis and glycogenolysis in the liver are presented in Fig. 3. Liver *Ppargc1a*, *Creb3l3* and *Pck1* increased (*P* < 0⋅0001 for all) and *Pygl* tended to increase (*P* = 0⋅057) following exercise compared to rest. Liver *Ppargc1a* more than doubled at 1 h post-exercise compared to rest [3⋅22 (1⋅34, 5⋅10), *P-*adjusted = 0⋅0002] and returned to rest at 3 h [−3⋅48 (−5⋅48, −1⋅48), *P*-adjusted = 0⋅0002 compared to 1 h; −0⋅264 (−2⋅05, 1⋅52) *P*-adjusted = 0⋅98 compared to rest]. At 6 h post-exercise, liver *Ppargc1a* decreased compared to 1 h [−2⋅02 (−4⋅02, −1⋅48), *P*-adjusted = 0⋅048], but was not different from any other timepoints (*P*-adjusted > 0⋅18 for all). Liver *Creb3l3* was not different a 1 h post-exercise [0⋅254 (−0⋅385, 0⋅894), *P*-adjusted = 0⋅72] but increased by 67% at 3 h [0⋅761 (0⋅138, 1⋅38), *P*-adjusted = 0⋅011] and 139% at 6 h [1⋅58 (0⋅955, 2⋅20), *P*-adjusted < 0⋅0001] compared to rest. Liver *Pck1* increased 1-fold at 1 h [2⋅18 (0⋅460, 3⋅91), *P*-adjusted = 0⋅0079] and nearly 2-fold at 6 h [3⋅61 (1⋅93, 5⋅29), *P*-adjusted < 0⋅0001] but was not different at 3 h [0⋅521 (−1⋅16, 2⋅20), *P*-adjusted = 0⋅84] compared to rest. Except for liver *Creb3l3*, which was not different between genotypes [0⋅124 (−0⋅231, 0⋅479), *P* = 0⋅48], all other markers of gluconeogenesis and glycogenolysis were greater in REDD1 KO mice compared to WT mice [*Ppargc1a*: 1⋅10 (0⋅159, 2⋅04), *P* = 0⋅0078; *Pck1*: 1⋅30 (0⋅399, 2⋅20), *P* = 0⋅011; *Pygl*: 0⋅408 (0⋅199, 0⋅618), *P* = 0⋅0005].

**Fig. 4.**
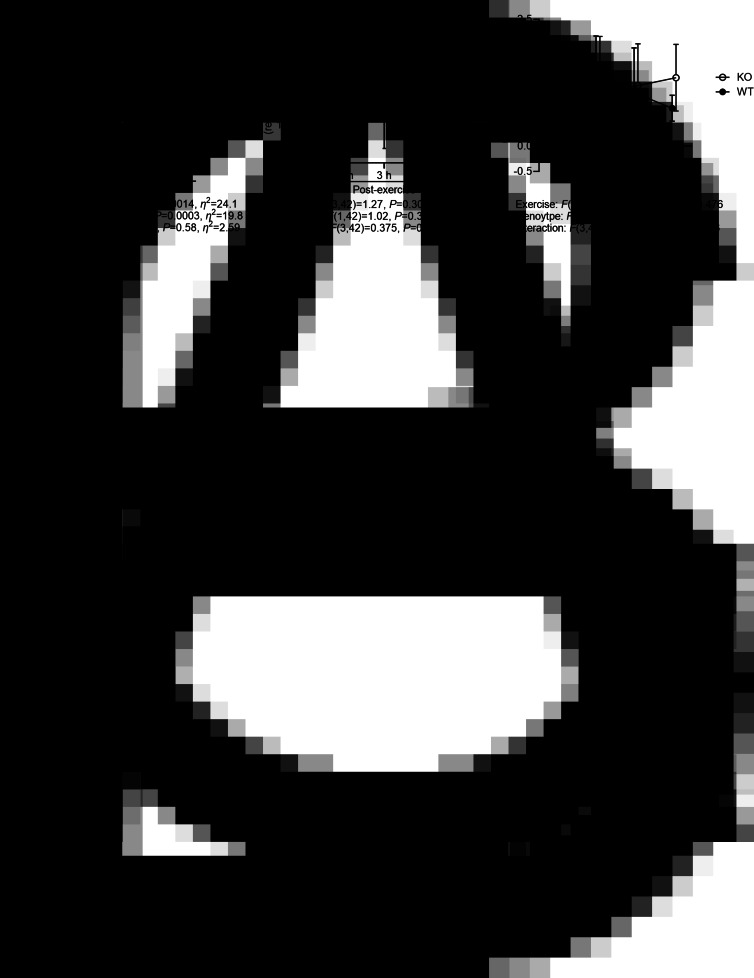
Liver gene expression of inflammatory markers in REDD1 KO and WT mice following rest or 1, 3 or 6 h recovery from 60 min treadmill running. Gene expression is fold change relative to rested WT mice. Data were analysed using two-way analyses of variance. *Orm1* data were log-transformed for analysis. Samples sizes are *n =* 8 per group for rested mice and *n =* 5–6 per group for exercised mice, except *Crp* and *Orm1* expression were not measured in one rested WT animal. Main effects of exercise, genotype and their interaction are presented as: *F*-value (degrees of freedom), *P*-value and *η*^2^. Data are presented as untransformed means (95% CIs). KO, knockout; REDD1, regulated in development and DNA damage response-1; WT, wild-type.

Markers of inflammation in the liver are presented in Fig. 4. Main effects of genotype (*P* = 0⋅0003) and exercise (*P* = 0⋅0014) were observed for liver *Crp*. Liver *Crp* was 33% greater in REDD1 KO mice compared to WT mice [0⋅428 (0⋅207, 0⋅650)]. Liver *Crp* increased 40% at 3 h post-exercise [0⋅458 (0⋅00875, 0⋅906), *P*-adjusted = 0⋅044] and 51% at 6 h post-exercise [0⋅590 (0⋅141, 1⋅04), *P*-adjusted = 0⋅0055] compared to rest. No main or interaction effects were observed for liver *Orm1* (*P* ≥ 0⋅30 for all). Liver *Il6* was detected in 35 of 51 samples (69%) with relatively equal distribution of expression across genotypes and exercise groups; there were no main or interaction effects (*P* ≥ 0⋅50 for all). *Saa1* was not detected in liver, except for in a few mice post-exercise which corresponded with elevated liver *Crp* (data not shown).

**Fig. 5.**
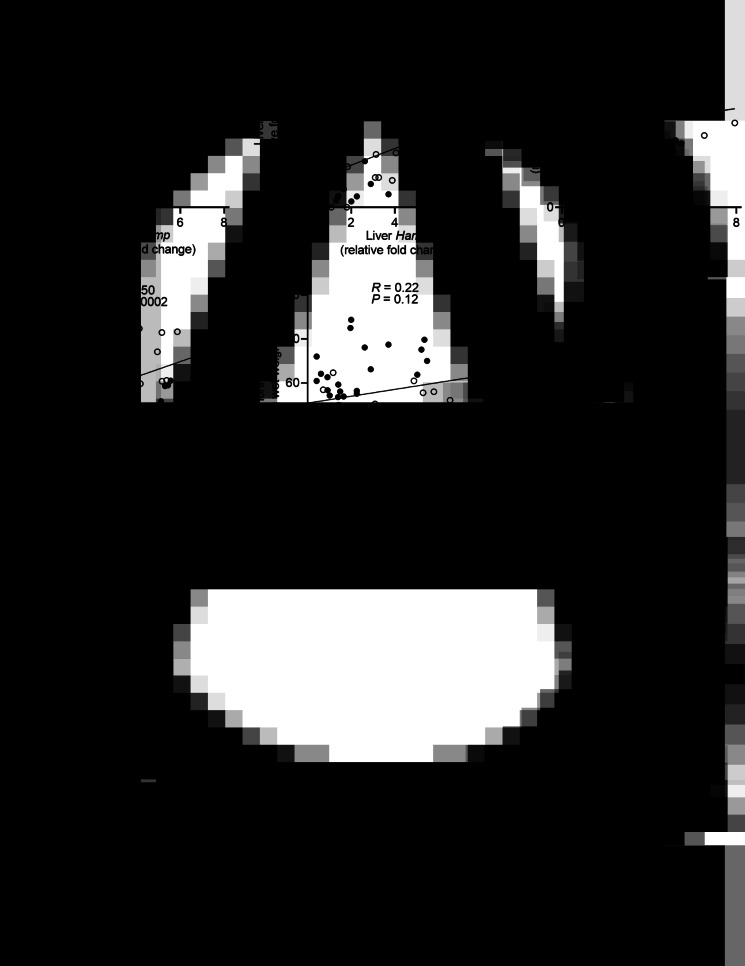
Correlations between liver *Hamp* and liver gene expression and liver non-heme iron in REDD1 KO and WT mice after rest or 1, 3 and 6 h recovery from 60 min treadmill running. Gene expression is fold change relative to rested WTmice with β-actin as the housekeeper. Data were analysed with Pearson correlations, and all variables were log-transformed prior to analysis except *Pygl* and liver non-heme iron. Samples sizes are *n =* 8 per group for rested mice and *n =* 5–6 per group for exercised mice, except *Crp* expression was not measured in one rested WT animal. Data are presented untransformed. KO, knockout; REDD1, regulated in development and DNA damage-1; WT, wild-type.

Correlations between liver *Hamp* and other outcomes collapsed across timepoints and genotypes are shown in Fig. 5, and correlations within each genotype are shown in Supplementary Table S4. Liver *Hamp* was positively correlated with liver *Creb3l3* (*R* = 0⋅56, *P* < 0⋅0001), *Pck1* (*R* = 0⋅44, *P* = 0⋅0014) and *Crp* (*R* = 0⋅50, *P* = 0⋅0002). In most cases, liver *Hamp* was positively correlated with each of these genes in REDD1 KO mice but not WT mice (Supplementary Table S4). Liver *Crp* was the exception, where there were positive correlations with liver *Hamp* in both genotypes. Neither non-heme iron in the liver (*R* = 0⋅22, *P* = 0⋅12) nor spleen (*R* = –0⋅14, *P* = 0⋅41) were correlated with liver *Hamp* when assessed in genotypes combined; however, liver *Hamp* was positively correlated with liver non-heme iron in REDD1 KO mice (*ρ* = 0⋅41, *P* = 0⋅046) and WT mice (*ρ* = 0⋅54, *P* = 0⋅0042), and liver *Hamp* trended towards a negative correlation with spleen non-heme iron in WT mice (*ρ* = –0⋅40, *P* = 0⋅050). Correlations were also assessed between other genes in the liver. Liver *Redd1* was positively correlated with liver *Ppargc1a* (*ρ* = 0⋅79, P < 0⋅0001), *Pck1* (*ρ* = 0⋅72, *P* < 0⋅0001) and *Pygl* (*ρ* = 0⋅39, *P* = 0⋅049) but was not correlated with any other outcomes (*P* ≥ 0⋅20 for all), and liver *Creb3l3* was positively correlated with liver *Crp* (*R* = 0⋅41, *P* = 0⋅0029). All relations were maintained when excluding rested mice, except liver *Creb3l3* was no longer correlated with liver *Crp* (*P* = 0⋅40).

## Discussion

The present study assessed the effects of a prolonged bout of running and REDD1 deletion on hepcidin and its upstream regulators in 12-week-old male B6/129F1 mice. The primary finding was that both exercise and REDD1 deletion increased expression of liver *Hamp* and genes indicating hepatic gluconeogenesis (*Ppargc1a*, *Pck1*), glycogenolysis (*Pygl*) and inflammation (*Crp*). The increase in liver *Hamp* with exercise corresponded with greater liver non-heme iron concentrations at 6 h post-exercise, suggesting a physiologic effect of hepcidin. These findings support the large amount of literature demonstrating an increase in hepcidin 3–6 h after a prolonged bout of exercise, which may factor into the declines in iron status frequently reported in physically active populations^(13,14)^.

Vecchi *et al.*^(5)^ first reported that acutely starved mice have increased liver hepcidin gene and protein expression, reduced liver ferroportin protein and hypoferremia compared to mice fed *ad libitum*. Moreover, *Creb3l3* KO mice were unable to up-regulate hepcidin and did not become hypoferremic during acute starvation. The authors then identified constitutive binding of PGC1α and CREB3L3 on the hepcidin promoter region in human and mouse liver cell cultures under gluconeogenic stimuli (glucagon, cAMP), providing a mechanism of hepcidin induction by gluconeogenic signals. In the present study, liver *Ppargc1a*, *Creb3l3* and *Pck1* increased with exercise and liver *Hamp* was positively correlated with liver *Creb3l3* and *Pck1*, suggesting that gluconeogenic signalling of hepcidin through the PGC1α–CREB3L3 mechanism may contribute to the increase in hepcidin following a bout of prolonged exercise.

The increase in liver *Hamp* expression with exercise tended to be greater in REDD1 KO mice, however the interaction was not statistically significant (*P* = 0⋅080). Particularly in REDD1 KO mice, liver *Hamp* was positively correlated with gene expression of gluconeogenic and glycogenolytic enzymes, suggesting heightened gluconeogenesis in the REDD1 KO mice. PGC1α is thought to stabilise CREB3L3 on the hepcidin gene promoter^(5)^. The greater liver *Ppargc1a* expression with REDD1 deletion in the present study may be driving the genotypic difference in liver *Hamp*, whereby PGC1α provides increased stabilisation of CREB3L3 protein on the hepcidin promoter in REDD1 KO mice despite no difference in *Creb3l3* expression between genotypes. Greater PGC1α and CREB3L3 activity is supported by greater hepatic gene expression of *Pck1* and *Pygl* with REDD1 deletion and greater *Pck1* expression with exercise, indicating transcriptional promotion of hepatic gluconeogenesis and glycogenolysis by PGC1α and CREB3L3^(30)^. These data further support the role of gluconeogenesis in the hepcidin response and reduced dietary iron absorption following a bout of prolonged exercise^(8,11,15)^ and indicate the glucoregulatory role of REDD1 and its possible impact on iron metabolism. Interestingly, liver *Ppargc1a* and *Pck1* expression increased at 1 h post-exercise, and both trended downwards at 3 h and appeared to increase again by 6 h post-exercise. A similar biphasic pattern of REDD1 expression occurred in the liver and has been shown in muscle following prolonged running^(31)^, which may be attributable to regulation of REDD1 by glucocorticoids^(17)^. As *Pygl* expression remained constant, the decrease in gluconeogenic gene expression at 3 h post-exercise may indicate a greater reliance on hepatic glycogenolysis. The secondary increase in gluconeogenesis at 6 h may be in response to the cumulative effects of the pre-exercise 3 h fast, exercise bout, and fasted rest period following exercise (10 h fast in total).

Liver *Crp* expression increased with exercise and was positively correlated with liver *Hamp*, supporting a hepcidin response during the acute phase response following a prolonged bout of running^(13)^. Somewhat surprisingly, there was greater liver *Crp* in REDD1 KO mice compared to WT mice. This contrasts with previous literature where the inflammatory response to acute challenges is reduced with REDD1 deletion^(32,33)^ and may indicate differing roles for REDD1 during the acute phase response depending on the tissue and/or the inflammatory challenge. In the present study, liver *Il6* was not impacted by exercise or REDD1 deletion most likely due to the timing of tissue collection (1, 3 and 6 h following exercise). In rodents and humans, IL-6 peaks in circulation immediately following prolonged exercise and rapidly declines^(15,16)^. Similar increases immediately following exercise have been observed for IL-6 gene expression in liver^(34)^ and muscle^(35–37)^, however post-exercise time course data for gene expression is limited. Considering the data on circulating IL-6, it seems likely that the timepoints for tissue analysis in the present study missed the peak in IL-6 immediately following exercise. Future studies on REDD1 and hepcidin should assess the time course of IL-6 in liver, muscle and circulation, including during and immediately following prolonged exercise. Moreover, circulating IL-6 stimulates hepatic gluconeogenesis following prolonged exercise but can have differing effects depending on the tissue source of IL-6^(34)^. Thus, future work should explore whether liver and muscle-derived IL-6 have discriminate effects on gluconeogenic and JAK–STAT signalling of hepcidin.

Liver non-heme iron was greater at 6 h recovery, compared to rest, indicating iron sequestration due to the hepcidin response to exercise^(3,4)^. Baseline iron status is a known contributor to post-exercise hepcidin, whereby circulating hepcidin following prolonged exercise is greater with increased baseline serum ferritin and serum iron in humans^(38,39)^. In the present study, liver non-heme iron was lower in REDD1 KO mice compared to WT mice, when assessed in the entire cohort and when using an unpaired *t*-test between rested WT and REDD1 KO mice (*P* = 0⋅042). Despite differences in iron stores, the increase in liver non-heme iron at 6 h recovery compared to rest appeared similar between genotypes (30% for WT mice and 32% for KO mice) and may suggest that the genotypic differences in post-exercise *Hamp* are not physiologically meaningful. REDD1 deletion could decrease iron stores through other mechanisms. REDD1 is known to contribute to regulation of circadian clock genes^(23)^, so it is possible that REDD1 deletion resulted in greater diurnal increases in hepcidin to decrease dietary iron absorption and basal iron status^(40)^.

The present study has strengths in measuring a broad array of genes related to iron homeostasis, energy metabolism and inflammation; however, there are limitations. Because this was a secondary analysis using tissues from experiments conducted by Gordon *et al.*^(21)^, the study was not initially designed to study iron metabolism. For instance, it is important to note that mice in this study were fed chow diets containing ~200 mg/kg iron. High concentrations of dietary iron have been shown to increase liver iron stores and mask the hepcidin response to iron-loading and inflammatory agents^(41)^, suggesting that the increase in hepcidin with exercise in the present study is robust. As a secondary analysis, the study was limited by tissue availability, and thus future studies should confirm our results by assessing endogenous substrate status and circulating markers of iron status, gluconeogenesis and inflammation. With both REDD1 deletion and exercise, we would expect increases in circulating hepcidin, gluconeogenic precursors and IL-6 and decreases in serum iron and muscle/liver glycogen. Furthermore, future studies should assess hepatic signalling cascades that promote hepcidin in the liver. We expect both REDD1 deletion and exercise to increase hepatic JAK–STAT signalling and PGC1α–CREB3L3 promotion of hepcidin. Studies with such outcomes may provide stronger evidence for causality, as our study is limited by findings that are largely associative. Finally, these findings should be further explored in female mice and using different mouse strains, as hepcidin and iron metabolism can differ by sex^(15,42,43)^ and strain^(44,45)^.

In conclusion, the present study demonstrates increases in hepcidin with exercise and REDD1 deletion and provides evidence for gluconeogenic signalling of hepcidin following prolonged exercise. These findings may inform nutritional interventions to prevent increases in hepcidin, such as periodised caloric or carbohydrate supplementation around a bout of prolonged exercise. More broadly, these conclusions may support a role of gluconeogenic signalling of hepcidin in the decline in iron status frequently observed in physically active and undernourished populations.

## Supplementary material

For supplementary material accompanying this paper visit https://doi.org/10.1017/jns.2023.37. click here to view supplementary material
