# Supplementary material for: REDD1 deletion and treadmill running increase liver hepcidin and gluconeogenic enzymes in male mice
Source: J Nutr Sci. 2023 Apr 14;12:e49. doi: 10.1017/jns.2023.37 (PMC10131055; doi:10.1017/jns.2023.37)
Supplement: Supplementary file 1 [file S204867902300037Xsup001.docx]

**Supplemental Table 1.** TaqMan primer-probe sets for qRT-PCR ^a^.

| **Gene name** | **Protein name** | **Assay product number** |
| --- | --- | --- |
| **Primary outcome** | | |
| *Hamp* | Hepcidin | Mm04231240_s1 |
| **Gluconeogenic regulators of *Hamp*** | | |
| *Ppargc1a* | PGC1α | Mm01208835_m1 |
| *Creb3l3* | CREB3L3 | Mm00520279_m1 |
| **Gluconeogenic and glycogenolytic enzymes** | | |
| *Pck1* | PEPCK | Mm01247058_m1 |
| *Pygl* | Liver glycogen phosphorylase | Mm01289790_m1 |
| **Inflammatory markers** | | |
| *Il6* | Interleukin-6 | Mm00446190_m1 |
| *Crp* | C reactive protein | Mm00432680_g1 |
| *Orm1* | α-1-acid glycoprotein | Mm00435456_g1 |
| *Saa1* | Serum amyloid A1 | Mm00656927_g1 |
| **Erythropoietic signals** | | |
| *Erfe* | Erythroferrone | Mm00557748_m1 |
| **Housekeeper gene** | | |
| *Actb* | β-actin | Mm02619580_g1 |

^a^ CREB3L3, hepatic-specific cAMP response element binding protein-3-like-3; PEPCK, phosphoenolpyruvate carboxykinase; PGC1α, peroxisome proliferator-activated receptor-γ coactivator-1α; REDD1, regulated in development and DNA damage response-1; qRT-PCR, quantitative real-time polymerase chain reaction.

**Supplemental Table 2.** *Redd1* primer sequences for qRT-PCR with PowerUp SYBR Green Master Mix ^a^.

| **Forward primer** (5’-3’) | **Reverse primer** (3’-5’) | **Amplicon size** (bp) |
| --- | --- | --- |
| TGGTGCCCACCTTTCAGTTG | GTCAGGGACTGGCTGTAACC | 121 |

^a^ *Redd1* primers were previously reported by Gordon et al. [21]. REDD1, regulated in development and DNA damage response-1; qRT-PCR, quantitative real-time polymerase chain reaction.

**Supplemental Table 3.** Power analysis input and output to estimate required sample size for 95% power to detect an effect of treadmill running on liver *Hamp* expression in mice ^a^.

| **F tests - ANOVA: Fixed effects, omnibus, one-way** | | | |
| --- | --- | --- | --- |
| **Analysis:** | A priori: Compute required sample size | | |
| **Input:** | Effect size f | = | 1.076453 |
|  | α err prob | = | 0.05 |
|  | Power (1-β err prob) | = | 0.95 |
|  | Number of groups | = | 4 |
| **Output:** | Noncentrality parameter λ | = | 23.175 |
|  | Critical F | = | 3.238872 |
|  | Numerator df | = | 3 |
|  | Denominator df | = | 16 |
|  | Total sample size | = | 20 |
|  | Actual power | = | 0.959038 |

^a^ Power analysis was performed in G*Power version 3.1 (32). Effect size was determined using an F-statistic derived from estimated means ± SDs from Banzet et al. (30).

**Supplemental Table 4.** Spearman correlations between liver *Hamp* and other outcomes assessed in REDD1 KO and WT mice combined and separately ^a^.

|  | **KO + WT**  (*n* = 51) | **WT**  (*n =* 26) | | **KO**  (*n* = 25) | |
| --- | --- | --- | --- | --- | --- |
| **Primary outcome** | | | | | |
| *Redd1* | -- | | -0.083 | | -- |
| **Gluconeogenic regulators of *Hamp*** | | | | | |
| *Ppargc1a* | 0.078 | | 0.0024 | | –0.17 |
| *Creb3l3* | 0.56*** | | 0.34 | | 0.71*** |
| **Gluconeogenic and glycogenolytic enzymes** | | | | | |
| *Pck1* | 0.42** | | 0.24 | | 0.43* |
| *Pygl* | 0.27 | | –0.019 | | 0.25 |
| **Inflammatory markers** | | | | | |
| *Crp* | 0.57*** | | 0.41* | | 0.62** |
| *Il6* | 0.018 | | –0.15 | | 0.15 |
| *Orm1* | 0.15 | | –0.019 | | 0.30 |
| **Tissue nonheme iron** | | | | | |
| Liver nonheme iron | 0.22 | | 0.54** | | 0.41* |
| Spleen nonheme iron | –0.14 | | –0.40 | | 0.12 |

^a^ Correlations were assessed with liver *Hamp* as the dependent variable and are expressed as Spearman’s *ρ*. Samples sizes are *n* = 8 per group for rested mice and *n* = 5-6 per group for exercised mice, except *Crp, Orm1*, and spleen nonheme iron were not measured in one rested WT animal. KO, knockout; REDD1, regulated in development and DNA response-1; WT, wildtype. *indicates 0.05 > *P* ≥ 0.01. **indicates 0.01 > *P* ≥ 0.001. ***indicates *P* < 0.001.
